# Are young children able to learn exploratory strategies by observation?

**DOI:** 10.1007/s00426-017-0896-0

**Published:** 2017-07-20

**Authors:** Francesca Foti, Domenico Martone, Stefania Orrù, Simone Montuori, Esther Imperlini, Pasqualina Buono, Laura Petrosini, Laura Mandolesi

**Affiliations:** 1 Department of Medical and Surgical Sciences, University “Magna Graecia”, Catanzaro, Italy; 2 IRCCS Fondazione Santa Lucia, Via del Fosso di Fiorano 65, 00143 Rome, Italy; 3 Department of Movement Sciences and Wellbeing, University “Parthenope”, Naples, Italy; 4 Fondazione IRCCS SDN, Naples, Italy; 5 Department of Psychology, “Sapienza” University of Rome, Rome, Italy; 6 Department of Motor Science and Wellbeing, University “Parthenope”, Via Medina, 40, 80133 Naples, Italy

## Abstract

New competencies may be learned through active experience (experiential learning or learning by doing) or observation of others’ experiences (learning by observation). Observing another person performing a complex action facilitates the observer’s acquisition of the same action. The present research is aimed at analyzing if the observation of specific explorative strategies adopted in a constrained environment, such as the Radial Arm Maze (RAM), could help young children to explore the maze and to build a cognitive spatial map of the explored environment. To this aim young children were randomly assigned to three groups: children who performed the RAM task following the observation of an actor solving the same maze by putting into action a highly structured exploratory strategy; children who performed the RAM task following the observation of the actor solving the same maze by putting into action a less structured exploratory strategy; children who directly performed the RAM task without any observation. The main result of the present research is that the children who observed the highly structured and correct exploratory strategy spent less time, made fewer errors, exhibited a longer spatial span, and thus they explored the maze more efficiently than the children who directly performed the RAM task without any observation. This finding indicates that when the observed explorative procedure is structured, sequential and repetitive the action understanding and information storage processes are more effective. Importantly, the observation of specific spatial strategies helped the children to build the cognitive spatial map of the explored environment and consequently to acquire/enrich the declarative knowledge of the environment.

## Introduction

New competencies may be learned through active experience (experiential learning or learning by doing) or observation of others’ experiences (learning by observation) (Bandura, 1977; Meltzoff, Kuhl, Movellan, & Sejnowski, 2009).

Learning by observation does not just involve copying an action, but it requires that the observer transforms the observation into an action as similar as possible to the model in terms of the goal to be reached and motor strategies to be applied (Meltzoff & Andrew, 1995; Meltzoff & Decety, 2003). Observing another person performing a complex action represents a desirable condition of learning that enables the learner to better understand the skill prior to the performance and/or it helps the learner to more readily discriminate perceptually variables that are important for the performance of that skill (Bird & Heyes, 2005; Meltzoff et al., 2009). It is believed that observation of an action facilitates motor learning of that skill because it facilitates the acquisition of the main spatial and temporal features of the task, and thus removes the need to create a cognitive representation of the action pattern through experiential learning (Keetch, Schmidt, Lee, & Young, 2005; Buchanan & Dean, 2010; Rohbanfard & Proteau, 2011). However, it is worth of noting that conditions of learning that accelerate the learning, by limiting the time-consuming process of learning by trial and error and reducing the practice needed to learn, often fail to support long-term retention and transfer (Schmidt & Bjork, 1992; Bjork, 2011; Bjork, Dunlosky, & Kornell, 2013).

Acquiring skills by observation is a fundamental cognitive ability already existing from the birth (Meltzoff & Moore, 1977; Nadel & Butterworth, 1998; Meltzoff et al., 2009; Nadel, 2002). Already at 18-months-old children may learn a novel motor pattern by observation (Herold & Akhtar, 2008; Matheson, Moore, & Akhtar, 2013) and if the adults explicitly show their intention prior to demonstration, even 16-months-old infants learn by observation (Fagard, Rat-Fischer, Esseily, Somogyi, & O’Regan, 2016). Three-year-old children are able to learn how to extract a reward from a box following a video-demonstration of the correct procedure (Flynn & Whiten, 2013).

Besides imitative abilities learning by observation requires cognitive competencies, as attentive and mnesic functions, sequencing abilities, planning, response inhibition, cognitive flexibility, good knowledge and anticipatory expectation of effects related to actions, goal-directed actions, and motor imagery allowing recombination of novel actions with novel effects (Foti et al., 2013, 2014, 2015; Torriero, Oliveri, Koch, Caltagirone, & Petrosini, 2007). Furthermore, to learn by observation it is necessary to observe and attend to the actor, engage in joint attention, understand and reproduce other’s actions. Thus, learning by observation also represents a powerful social learning mechanism (Frith & Frith, 2012). For example, children can learn how to behave in social contexts by observing how adults interact with each other (Shimpi, Akhtar, & Moore, 2013). Recently, it was shown that if the model has a high social status, such as a teacher, the children tend to learn even irrelevant information by observation (McGuigan, Gladstone, & Cook, 2012; McGuigan, 2013) or attempts without outcome (Carr, Kendal, & Flynn, 2015). The typical scenario in these studies is that before being allowed to attempt the task themselves, the observers watch an adult model perform a sequence of tool actions varying according to their causal necessity, with some of the actions being necessary for reward retrieval, others being causally irrelevant (as performing unnecessary taps before retrieving a reward from a box) and others without the efficacy of an observed solution. The findings of these researches suggest that young children are selective copiers who reproduce the irrelevant tool actions most frequently after having viewed high-status models performing them. Thus, learning by observation represents a learning mechanism that can be used in several fields (e.g., school and sport) as a “learning technique”. In addition, several studies have highlighted the importance of the observational learning in children with intellectual disabilities (Foti et al., 2013, 2014, 2015).

As for its neurobiological basis, the learning by observation is thought to utilize brain regions responsive to both observation and execution of action, as the mirror neuron system (Gallese, Fadiga, Fogassi, & Rizzolatti, 1996; Rizzolatti, Fogassi, & Gallese, 2001; Rizzolatti & Craighero, 2004). The mirror neuron system includes premotor cortex, inferior frontal gyrus, and inferior parietal lobule, areas which receive their main visual input from the superior temporal sulcus (Molenberghs, Brander, Mattingley, & Cunnington, 2010; Caspers, Zilles, Laird, & Eickhoff, 2010). Insofar as it generates a simulation circuit that allows the association between one’s own actions with others’ actions, the mirror neuron system is retained to be involved in action understanding, imagination, and imitation (Rizzolatti & Sinigaglia, 2010), and thus even in the observational learning.

Most developmental studies focused on how and what the observer child has to observe to promote learning (Rohbanfard & Proteau, 2011; Marshall & Meltzoff, 2014; Carr et al., 2015). However, to our knowledge there are no developmental studies that investigated whether the learning by observation of exploratory strategies promotes the acquisition of navigational abilities. For this reason, we wondered if observing an adult actor who adopts specific navigational strategies to explore a radial arm maze (RAM) can help young children improve their exploration of the same maze and build the cognitive spatial map of the explored environment. Another aim of the present research is to determine whether observation of a structured model or of a less structured model of explorative strategies would have resulted in different reproduction of the explorative patterns. On one hand, it has been proposed that the observation permits the observer to develop a sort of “perceptual blueprint” of the task to be learned (Bandura, 1977). This may work in favor of the utilization of a model performing a very efficient and successful explorative strategy. On the other hand, it might be fruitful also to observe a model performing a less structured and with a superior mnesic load, but still successful explorative strategy. To these aims, young children (mean age: 5 years and 3 months) were randomly assigned to three groups: children who performed the RAM task following the observation of an actor solving the same maze by putting into action a highly structured and efficient exploratory strategy (such as sequentially entering adjacent arms); children who performed the RAM task following the observation of the actor solving the same maze by putting into action a less structured and still successful exploratory strategy (such as randomly entering arms); children who directly performed the RAM task without any observational training.

## Methods

### Participants

Thirty-six healthy Italian children (17 M and 19 F) aged from 4 years and 6 months (4.6) to 5 years and 9 months (5.9) (mean age ± SD 5.3 ± 0.2) participated in the present study. Children were subdivided into three groups according to the following experimental conditions: Learning by Observation of a highly Structured explorative strategy (LeOS) (*N* = 11; 5 M and 6 F; mean age 5.2 ± 0.3); Learning by Observation of Random explorative strategy (LeOR) (*N* = 13; 6 M and 7 F; mean age 5.3 ± 0.2); Learning by Doing (LeD) (*N* = 12; 6 M and 6 F; mean age 5.3 ± 0.1). No children have had previous experience with the RAM task.

All children had normal or corrected-to-normal vision and standard anthropometric measurements and presented no neurological or neuropsychological problems. Body mass index values (*M* = 17.06 ± 2.8; *F* = 16.83 ± 1.62) were between the 50th–75th percentile. To exclude the presence of sensory-motor deficits, the psychomotor development of all children was evaluated through a battery of exercises of motor accuracy (Niederer et al., 2011). To verify graphic abilities and cognitive development, all children were assessed in the drawing test of the human figure (Machover, 1949). All children attended a kindergarten school in South Italy where a 1 h/day of physical activity was planned for 5 days/week. The parents of children gave informed written consent. The study was conducted according to the 1964 Declaration of Helsinki.

### Motor accuracy assessment

**Fig. 1:**
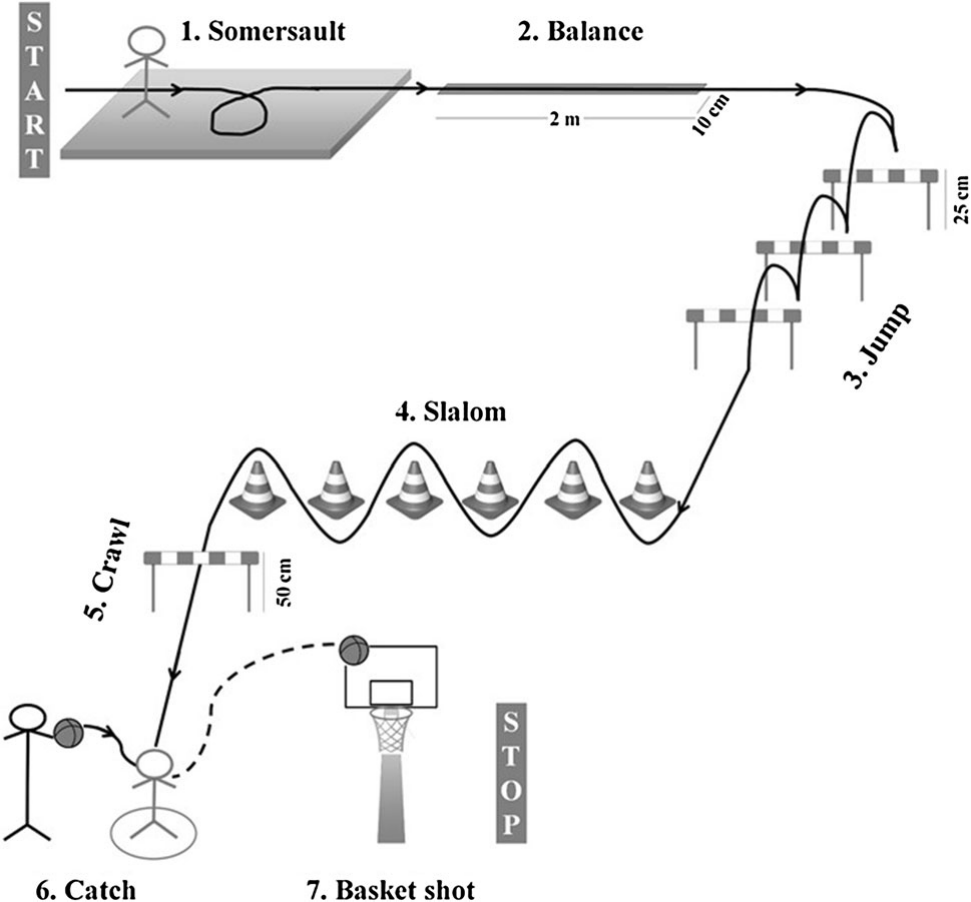
Schematic representation of the agility course

In the school gym an “agility course” was built (Fig. 1) where the following seven motor abilities were evaluated assigning “1” or “0” scores according to correctness or incorrectness, respectively.

*Somersaulting*: the child rolls forward in a complete revolution around the horizontal axis on a carpet at the start of the course; *Balancing*: the child walks heel to toe on a white 2 m (78.74 in) tape (10 cm (3.93 in) large) fixed on to the ground; *Jumping*: the child hops three 25 cm (9.84 in) high obstacles, built with two cones joined by a rod; *Slaloming*: the child runs in a zig zag pattern among six cones; *Crawling*: the child grovels under a rod held by two cones set at 50 cm (19.68 in) from the ground; *Catching*: the child enters a circle placed on the ground in which he/she grasps a ball thrown by the teacher positioned in front of him/her; *Shooting for goal*: the child throws the ball into the basket located in front of him/her.

*Total score* (the sum of scores ranging from 0 to 7) and *total time* (time to perform the entire course) were recorded.

### Drawing test of the human figure

According to Machover’s instructions (Machover, 1949), each child was asked to “draw someone”. For child’s question on what it was possible to draw, the experimenter replied “whatever you want”. If the child drew only the head, the investigator encouraged him/her to draw the whole figure. Since the children were less than 6 years of age, the qualitative assessment of drawing human figure was focused to highlight whether the child did not draw significant details, such as hands, hair, eyes, mouth (Di Leo, 1970; Cox, 1992; Boncori, 2006).

### Apparatus

**Fig. 2:**
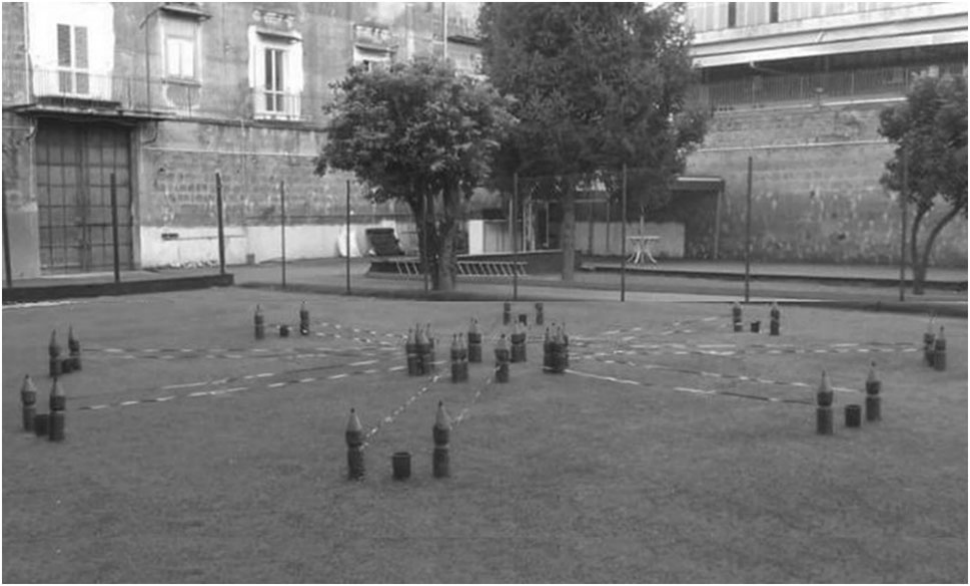
View of the eight-arm radial maze

The RAM adapted for children consisted of a round central platform [1 m (39.37 in) in diameter] with eight arms [50 cm (19.68 in) wide × 3.5 m (137.79 in) long] radiating like the spokes of a wheel (Fig. 2). To force the child to exit from an arm and return to the center of the starting platform before entering another arm, the sides of each arm were marked off by white and red ribbons hung across the opening and the end of the arm, forming a sort of constraining barrier. This procedure prevented the children from “cutting corners” as they exited from an arm and forced them to exit and return to the center of the starting platform before entering another arm. At the end of each arm, there was a red plastic bucket (18 cm (7.08 in) wide × 28 cm (11.02 in) high) containing the reward (a little colored ball). The RAM, located outdoors in a football field, was surrounded by extra-maze cues (trees, swings, benches, etc.) held in constant spatial relations throughout the experiment. The arms were virtually numbered in a clockwise direction, considering arm 1 as the farthest from the experimenter’s location. Only during the experiment could the children see the maze or have physical access to it. To increase the motivation of picking up the rewards, at the end of each trial the child received a reward (a little toy) in exchange for all the colored balls found in the buckets.

### Experimental procedure

The three experimental conditions were:—Learning by Observation of a highly Structured explorative strategy (LeOS), in which the children performed the RAM task following the observation of an actor solving the maze through a highly structured exploratory strategy;—Learning by Observation of Random explorative strategy (LeOR), in which the children performed the RAM task following the observation of the actor solving the maze through a less structured exploratory strategy;—Learning by Doing (LeD) in which the children directly performed the RAM task without observation (Table 1).

**Table 1:** Experimental procedures of the three experimental conditions

	Day 1	Day 2	Day 3 I session	Day 4 II session	Day 5 III session
LeD			RAM testing	RAM testing	RAM testing
LeOS					
Morning	Observation of: I trial: 1–2–3–4–5–6–7–8 II trial: 2–3–4–5–6–7–8–1 III trial: 3–4–5–6–7–8–1–2	Observation of: VII trial: 7–8–1–2–3–4–5–6 VIII trial: 8–1–2–3–4–5–6–7 IX trial: 1–2–3–4–5–6–7–8	RAM testing	RAM testing	RAM testing
Afternoon	Observation of: IV trial: 4–5–6–7–8–1–2–3 V trial: 5–6–7–8–1–2–3–4 VI trial: 6–7–8–1–2–3–4–5				
LeOR					
Morning	Observation of: I trial: 2–7–8–3–5–1–4–6 II trial: 8–3–2–5–7–4–1–6 III trial: 3–5–8–7–1–4–6–2	Observation of: VII trial: 1–6–2–5–4–7–3–8 VIII trial: 2–4–7–8–3–5–1–6 IX trial: 5–8–3–4–6–2–7–1	RAM testing	RAM testing	RAM testing
Afternoon	Observation of: IV trial: 6–8–3–4–7–2–5–1 V trial: 4–7–1–6–8–2–3–5 VI trial: 7–4–1–8–5–3–2–6				

The strings of numbers indicate the sequence of visited arms performed by the experimenter in the both conditions of learning by observation. Note that the experimenter explored the Radial Arm Maze entering only the adjacent arms in *Learning by Observation of a highly Structured explorative strategy* (LeOS), while he did not follow an evident navigational strategy in *Learning by Observation of Random explorative strategy* (LeOR)

In each trial of the RAM testing, each child was allowed to explore freely the eight arms to retrieve the reward. A trial ended when all eight rewards had been collected, 20 choices had been made, or 10 min had elapsed from the start of the task. Since the buckets were never rewarded twice, the optimal performance consisted of visiting each bucket only once. An error was made when the child re-entered an arm already visited during the same trial. Each child performed three trials a day for three consecutive days. Since the three daily trials constituted a session and each child made three sessions, each child performed nine trials. At the end of each trial, the child waited 1 h (inter-trial interval), before being re-tested in the RAM. At the beginning of RAM testing, the experimenter used the same simple verbal instructions to explain the task to each child (“The game is to find the little colored balls. Do you see the colored buckets at the end of each alley? You have to reach a bucket, take the little ball inside, and then go back to the center, where the platform is, until you have collected all the balls. Be careful to reach the buckets always staying inside the maze. Go and have fun!”). No other instructions or verbal encouragement were provided during testing. In the two observation conditions (LeOS and LeOR), before starting RAM exploration the experimenter told the children: “The game is to find the little colored balls inside the buckets. Look at me carefully”. In the LeOS condition, each child observed three sessions of three trials each in which the actor explored the RAM entering always the adjacent arms and stopped after the eight rewards were collected. In the LeOR condition, each child observed three sessions of three trials each in which the actor explored the RAM using a pseudorandom explorative strategy and stopping after eight rewards collected. In LeOS and LeOR conditions, children observed the actor at distance of about 1.5 m (59.05 in) from the RAM, changing their point of observation at every session. Then, each child actively experienced the three RAM sessions (RAM testing; Table 1). The trials were annulled if the child left the maze. However, very few children of LeD condition engaged in this behavior and, in any case, only in the very first trials of the task. In LeOS and LeOR conditions, no child left the maze. The RAM testing lasted 3 consecutive days and in this execution phase all children were videotaped and recorded manually. At the end of RAM testing phase, all children were asked to make a drawing of the setting where they had just “played” to evaluate their mental representative mapping abilities.

### Behavioral parameters

We evaluated:—*total time* (in seconds) spent to complete the task;—*entries*, calculated as the number of visited arms;—*errors*, calculated as the number of re-entries into already visited arms;—*spatial span*, calculated as the longest sequence of correctly visited arms;—*perseverations*, calculated as the percentage of consecutive entries into the same arm or the re-entries into a fixed sequence of arms, divided by the number of arms visited;—percentage of *angled turns*, calculated as the number of a given angle (45°, 90°, 135°, 180°, or 360°) the child made in each trial divided by the number of angles made × 100;—*declarative mastery*, calculated as the percentage of trials in which the child stopped the search after collecting the eight rewards as if he/she knew the task was finished.

In examining maze drawings, we evaluated the *type of representation*, an index rating the egocentricity/allocentricity of drawings using a 5-point Likert scale (from 1: clear egocentricity, to 5: clear allocentricity). To objectively assess this parameter in children’s drawings we asked a coder blind to RAM conditions and expert in mental spatial representations and human navigation to score each drawing according to its egocentricity/allocentricity.

### Statistical analyses

The data were first tested for normality (Shapiro–Wilk’s test) and homoscedasticity (Levene’s test). All data were presented as the mean ± SD and were analyzed by one- or two-way analyses of variance (ANCOVAs) with repeated measures (session/angle) and with age and gender as covariates followed by post hoc multiple comparisons when appropriate (Duncan’s test).

## Results

### Motor accuracy

All children similarly performed the agility course (total time 36.6 ± 2.2 s; total score 5.5 ± 1.3). Namely, almost all children failed in shooting for goal, an ability acquired relatively later, while all children successfully performed the slaloming. Table 2 shows the percentage of children who efficaciously performed each item of the motor accuracy task. A one-way ANCOVA failed to reveal any statistical difference among the three experimental groups in *total time* (*F*(2,31) = 0.28; *p* = 0.75; *η*_P_^2^ = 0.02) and *total score* (*F*(2,31) = 0.18; *p* = 0.98; *η*_P_^2^ = 0.001).

**Table 2:** Percentage of children of the three experimental conditions successfully performing each motor task of the agility course

	Somersaulting	Balancing	Jumping	Slaloming	Crawling	Catching	Shooting for goal
LeD	92	83	92	100	92	83	17
LeOS	91	91	82	100	100	73	18
LeOR	100	92	77	100	92	77	15

*LeD* Learning by Doing, *LeOS* Learning by Observation of a highly Structured explorative strategy, *LeOR* Learning by Observation of Random explorative strategy

### Drawing test of human figure

**Fig. 3:**
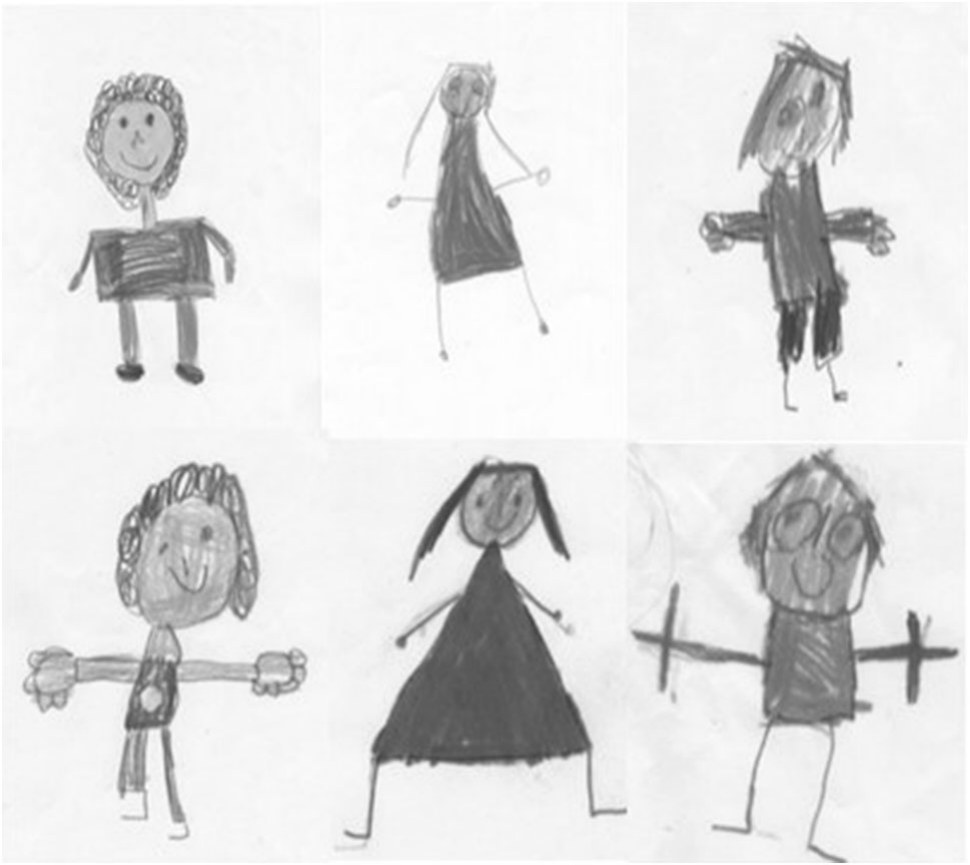
Drawings of human figure. Examples of drawings of children randomly selected among groups (Machover’s test**)**

Qualitative analysis of children’s drawings revealed that all children drew details of human figure in accordance with their age. All children drew many body parts, inserting the hands and the feet on to the arms and legs. Not only they drew the main body parts but they added more details including hairs and clothing features. Typically, the youngest children of our study used single lines and the oldest ones drew pairs of lines to represent arms and legs (Fig. 3). Their correct acquisition and internalization of the body image suggested a cognitive development appropriate for their age.

### Radial maze

#### Total time

**Fig. 4:**
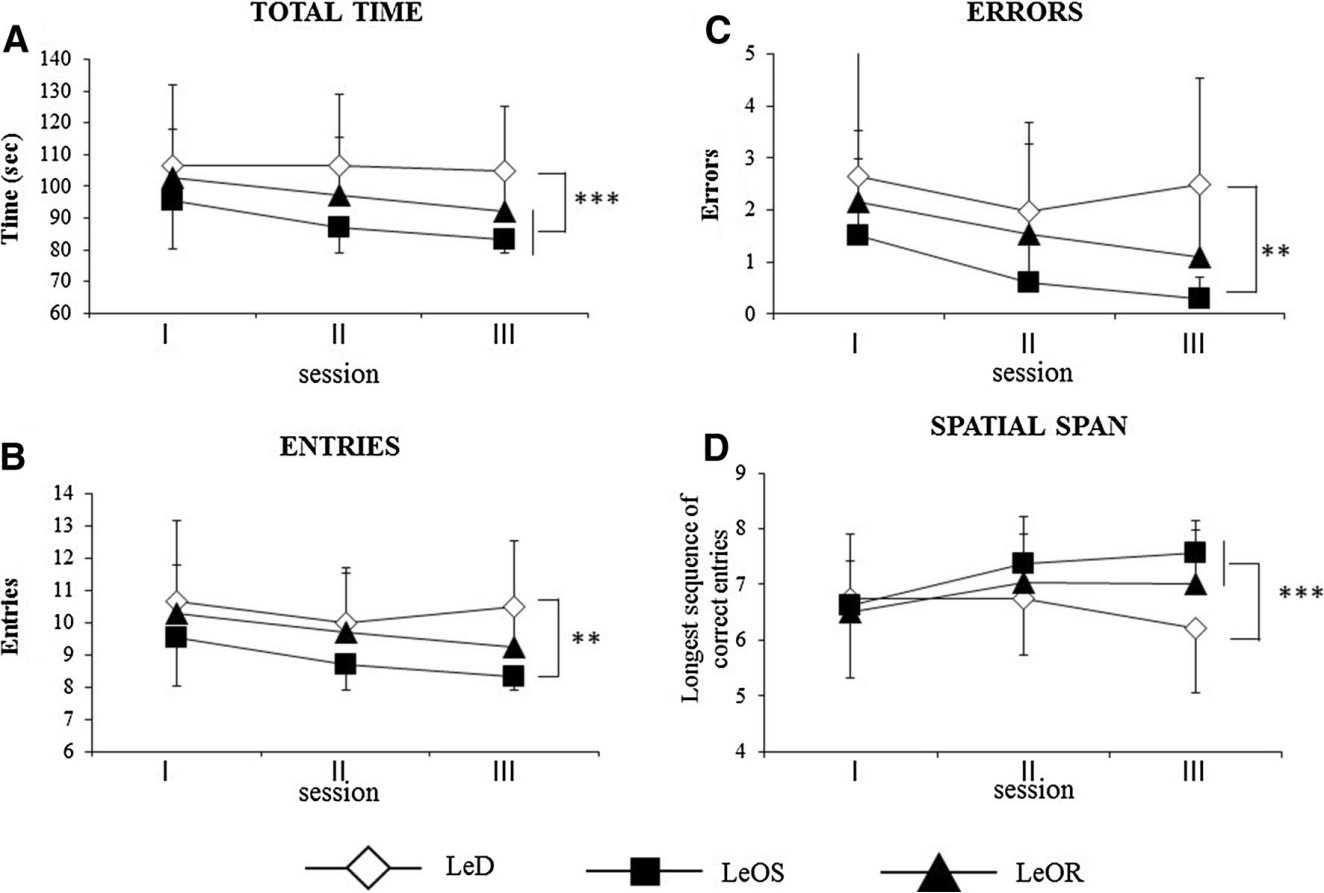
Performances in the Radial Arm Maze task. Data are expressed as mean ± SD. The *asterisks* indicate the significance level of post hoc comparisons among groups (*p < 0.05; **p < 0.01). In this and in the following figures: *LeD* Learning by Doing group, *LeOS* Learning by Observation of a highly Structured explorative strategy, *LeOR*, Learning by Observation of Random explorative strategy group

A two-way ANCOVA (group × session) revealed significant group (*F*(2,31) = 4.59; *p* = 0.01; *η*_P_^2^ = 0.23) and session (*F*(2,66) = 3.41; *p* = 0.04; *η*_P_^2^ = 0.09) effects, while the interaction was not significant (*F*(4,66) = 0.63; *p* = 0.64; *η*_P_^2^ = 0.04). Post hoc comparisons on group effect revealed that the children who had observed the actor (LeOS and LeOR groups) took less time than those belonging to LeD group (at least *p* < 0.04) (Fig. 4a; Table 3a).

**Table 3:** Post hoc comparisons of the significant factors of ANCOVAs

Behavioral parameter	Groups		
	LeD vs. LeOS *p*; Cohen’s *d*; *r*	LeD vs. LeOR *p*; Cohen’s *d*; *r*	LeOS vs. LeOR *p*; Cohen’s *d*; *r*
(a) Post hoc comparisons on the group effect of the two-way ANCOVAs			
Total time (mean of the 3 sessions)	***p*** **=** **0.0007** ***d*** **=** **0.99** ***r*** **=** **0.44**	***p*** **=** **0.04** ***d*** **=** **0.43** ***r*** **=** **0.21**	*p* = 0.09 *d* = −0.68 *r* = −0.32
Entries (mean of the 3 sessions)	***p*** **=** **0.006** ***d*** **=** **0.95** ***r*** **=** **0.42**	*p* = 0.21 *d* = 0.34 *r* = 0.17	*p* = 0.08 *d* = −0.70 *r* = −0.33
Errors (mean of the 3 sessions)	***p*** **=** **0.004** ***d*** **=** **0.99** ***r*** **=** **0.44**	*p* = 0.12 *d* = 0.43 *r* = 0.21	*p* = 0.11 *d* = −0.65 *r* = −0.31
Spatial span (third session)	***p*** **=** **0.0004** ***d*** **=** **−1.47** ***r*** **=** **−0.59**	***p*** **=** **0.04** ***d*** **=** **−0.72** ***r*** **=** **−0.34**	*p* = 0.12 *d* = 0.20 *r* = 0.10

Angle	Groups		
	LeD vs. LeOS *p*; Cohen’s *d*; *r*	LeD vs. LeOR *p*; Cohen’s *d*; *r*	LeOS vs. LeOR *p*; Cohen’s *d*; *r*
(b) Post hoc comparisons on the interaction of the two-way ANCOVA			
45°	***p*** **=** **0.0008** ***d*** **=** **−1.09** ***r*** **=** **−0.48**	*p* = 0.29 *d* = −0.28 *r* = −0.14	***p*** **=** **0.01** ***d*** **=** **0.74** ***r*** **=** **0.35**
90°	*p* = 0.13 *d* = 0.92 *r* = 0.42	*p* = 0.11 *d* = 1.2 *r* = 0.52	*p* = 0.87 *d* = 0.13 *r* = 0.07
135°	*p* = 0.81 *d* = 0.45 *r* = 0.22	***p*** **=** **0.04** ***d*** **=** **−1.28** ***r*** **=** **−0.54**	***p*** **=** **0.03** ***d*** **=** **−1.41** ***r*** **=** **−0.58**
180°	*p* = 0.19 *d* = 1.19 *r* = 0.51	*p* = 0.25 *d* = 0.93 *r* = 0.42	*p* = 0.82 *d* = −0.26 *r* = −0.13

Bold values are statistically significant

#### Entries

A two-way ANCOVA (group × session) revealed significant group (*F*(2,31) = 4.55; *p* = 0.01; *η*_P_^2^ = 0.22) and session (*F*(2,66) = 3.64; *p* = 0.03; *η*_P_^2^ = 0.09) effects, while the interaction was not significant (*F*(4,66) = 0.7; *p* = 0.59; *η*_P_^2^ = 0.04). Post hoc comparisons on group effect revealed that the children who had observed the actor solving the maze with a structured strategy (LeOS group) performed the task with a significantly lower number of entries in comparison to children who had never observed (LeD group) (*p* = 0.006). The children who had observed the actor solving the RAM with a random strategy (LeOR group) explored the maze making a number of entries similar to that of children belonging to LeD and LeOS groups (Fig. 4b; Table 3a).

#### Errors

A two-way ANCOVA (group × session) revealed significant group (*F*(2,31) = 5.29; *p* = 0.01; *η*_P_^2^ = 0.25) and session (*F*(2,66) = 3.85; *p* = 0.02; *η*_P_^2^ = 0.10) effects. The interaction was not significant (*F*(4,66) = 0.68; *p* = 0.60; *η*_P_^2^ = 0.04). Post hoc comparisons on group effect revealed that the children belonging to LeOS group made a significantly lower number of errors in comparison to children who had not observed (LeD group) (*p* = 0.004), while the children belonging to LeOR group made a similar number of errors to LeD and LeOS children (Fig. 4c; Table 3a).

#### Spatial span

A two-way ANCOVA (group × session) failed to reveal significant group (*F*(2,31) = 2.09; *p* = 0.14; *η*_P_^2^ = 0.12) and session (*F*(2,66) = 2.45; *p* = 0.09; *η*_P_^2^ = 0.07) effects, but the interaction was significant (*F*(4,66) = 2.52; *p* = 0.04; *η*_P_^2^ = 0.13). Post hoc comparisons on the interaction revealed that in the third session all children who had observed the actor (LeOS and LeOR groups) had span values significantly higher than children who directly experienced the maze (LeD group) (at least *p* < 0.04) (Fig. 4d; Table 3a).

#### Perseverations

No child performed consecutive entries into the same arm or into a fixed sequence of arms during RAM exploration.

#### Angle analysis

**Fig. 5:**
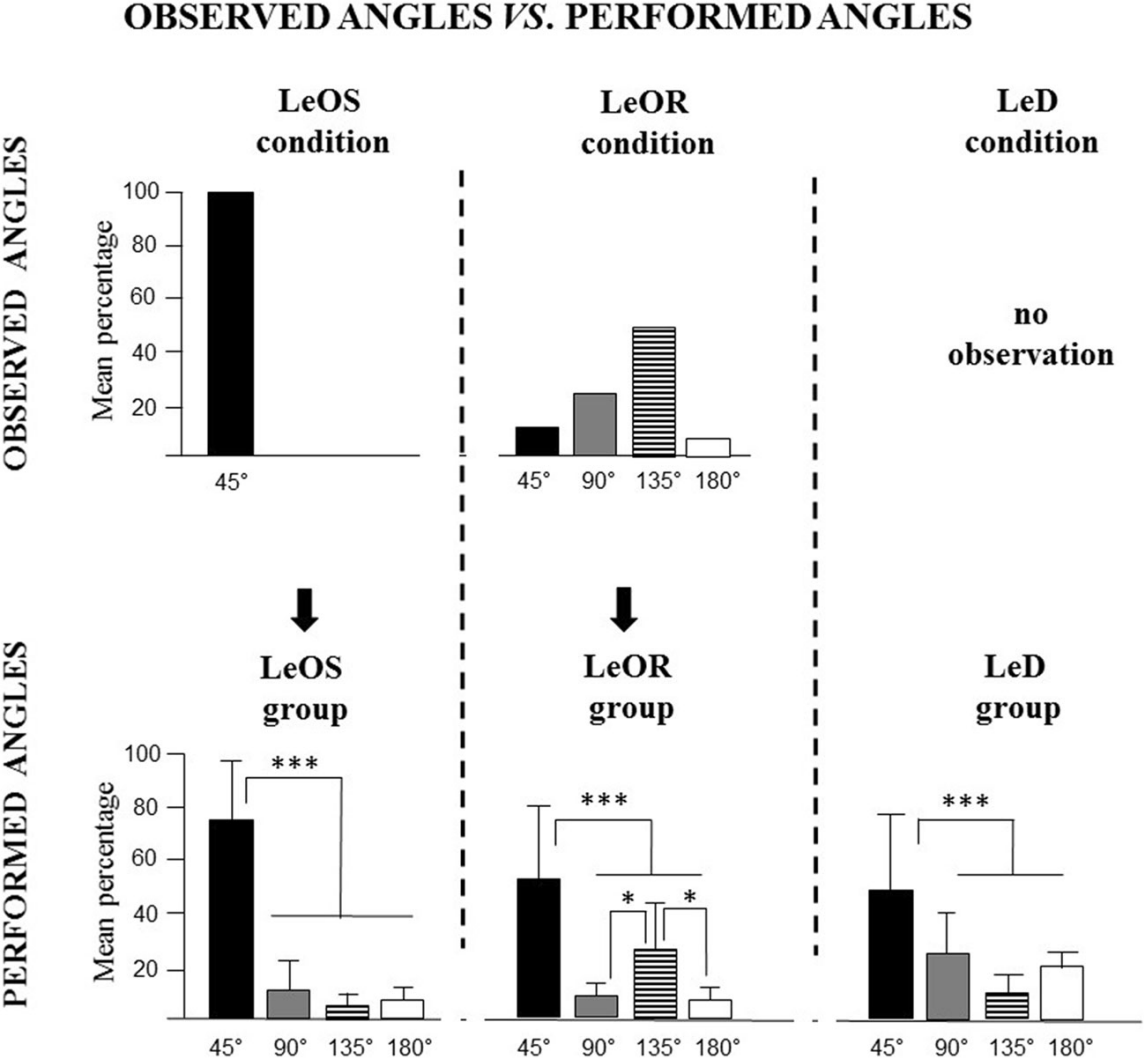
Observed angles vs. performed angles. Data are expressed as mean ± SD. The *asterisks* indicate the significance level of post hoc comparisons among groups (*p < 0.05; ***p < 0.001)

The angles performed in visiting RAM arms were closely linked to the navigational strategies put into action in exploring the maze. In the angle analysis, 360° angles are missing because no child performed them. The experimental procedure provided that the LeOS children observed the actor entering adjacent arms and making thus only 45° angles, while LeOR children observed the actor performing 45° (14% of total angles), 90° (25%), 135° (49%) and 180° (11%) angles (Fig. 5). A two-way ANCOVA (group × angle) failed to reveal a significant group effect (*F*(2,31) = 0.44; *p* = 0.65; *η*_P_^2^ = 0.03), while angle effect (F(3,99) = 43.67; *p* < 0.00001; *η*_P_^2^ = 0.57) and interaction (*F*(6,99) = 4.45; *p* = 0.0005; *η*_P_^2^ = 0.21) were significant. Interestingly, post hoc comparisons on interaction demonstrated that LeOS children obtained a significantly higher percentage (74%) of 45° angles in comparison to others groups (LeD 46%; LeOR 55%; at least *p* < 0.01), and LeOR children obtained a significantly higher percentage (26%) of 135° angles in comparison to others groups (LeD 7%; LeOS 6%; at least *p* < 0.046) (Fig. 5; Table 3b).

#### Declarative mastery

**Fig. 6:**
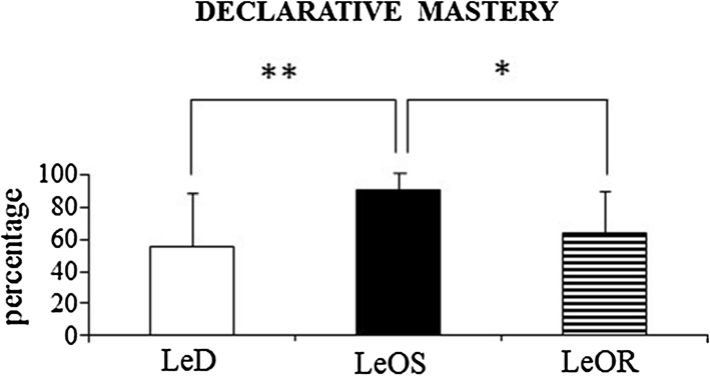
Declarative mastery. Data are expressed as mean ± SD. The *asterisks* indicate the significance level of post hoc comparisons among groups (**p < 0.0005)

A one-way ANCOVA was significant (*F*(2,31) = 5.75; *p* = 0.007; *η*_P_^2^ = 0.41). Post hoc comparisons (LeD vs. LeOS, *p* = 0.003; LeD vs. LeOR, *p* = 0.42; LeOS vs. LeOR, *p* = 0.02) demonstrated that LeOS children obtained a significantly higher percentage of declarative mastery in comparison to LeD and LeOR children (Fig. 6).

#### Drawing the maze

At the end of RAM testing, 12/12 LeD children, 11/11 LeOS children, and 8/13 LeOR children made a drawing of the setting where they had just played. The five uncooperative LeOR children who did not want to draw the maze were not forced to do it.

**Fig. 7:**
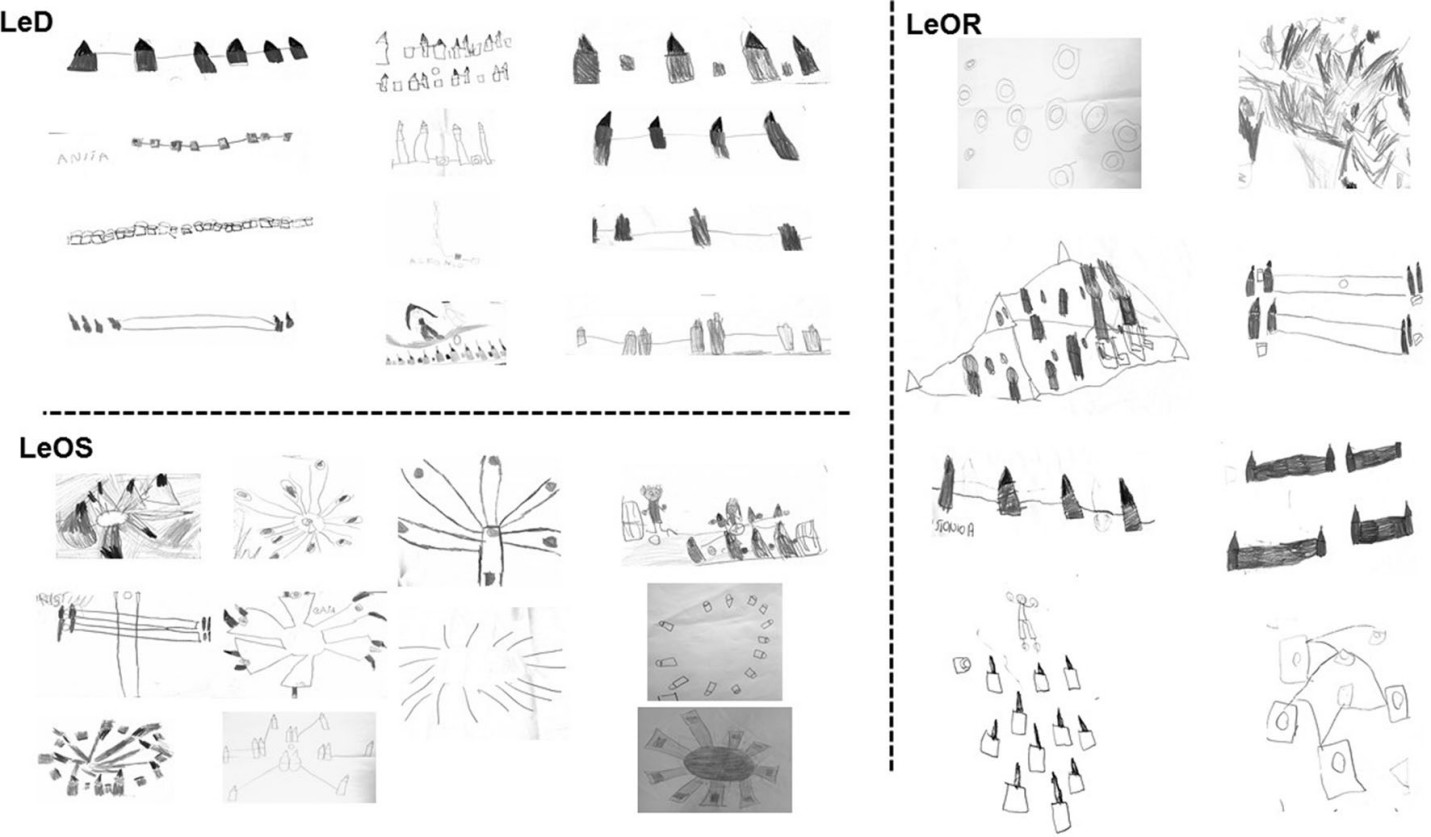
RAM representations. The drawings were made by the children of the three experimental groups at the end of the test

The type of representation of the experimental setting was significantly different among groups (one-way ANCOVA (*F*(2,26) = 41.36; *p* < 0.000001; *η*_P_^2^ = 0.31; Post hoc comparisons: LeD vs. LeOS, *p* = 0.00006; LeD vs. LeOR, *p* = 0.0004; LeOS vs. LeOR, *p* = 0.0002). In fact, the LeD children reached a mean score of 1.25 ± 0.45, indication that most of them drew the maze with an overtly egocentric representation. Conversely, the LeOS children reached a mean score of 4.64 ± 0.92. Interestingly, LeOR children reached a mean score of 2.88 ± 1.25, an intermediate score indicating that the observation of less structured navigational strategies did not allow building an allocentric representation of the environment (Fig. 7).

## Discussion

Learning by observation requires attentive and mnesic functions, sequencing and planning abilities, anticipatory expectation of effects, motor imagery, as well as engagement in joint attention, and understanding and reproducing other’s actions (Torriero et al., 2007; Menghini, Vicari, Mandolesi, & Petrosini, 2011; Foti et al., 2013, 2014, 2015). Although these abilities continue to mature throughout life, they are already present in pre-schoolers and young children (Mandolesi, Petrosini, Menghini, Addona, & Vicari, 2009a; Rohbanfard & Proteau, 2011; Marshall & Meltzoff, 2014; Carr et al., 2015).

Complex to-be-learned skills have generally an organizational structure that can be dissected into smaller units or types of behavior (i.e., extended or direct exploration) and the acquisition by observation of single exploratory strategies allows studying the learning power of specific behavioral units (Graziano et al., 2002). Conversely, a paradigm that involves actual experiential learning of explorative strategies renders almost impossible the singling out of single behavioral units. Starting from these premises, in the present study we singled out the observational learning of different explorative strategies adopted in a constrained environment, such as the RAM, and we analyzed if the observation of the single navigational strategies could promote the acquisition of navigational abilities and the building of the cognitive spatial map of the explored environment in young children.

The main result of the present research is that when the observed explorative procedure is structured, sequential and repetitive the action understanding and information storage processes are extremely effective. In fact, LeOS children made less entries and less errors, and reached values of spatial span significantly higher than LeD children. However, also the observation of an unstructured and random exploratory strategy facilitated RAM exploration. In fact, in comparison to LeD children, LeOR children took less time to end the trial and obtained higher span values. Interestingly, the strategy the children observed influenced their exploration, as indicated by angle analysis. While the LeOS children observed the actor performing only 45° angles, the LeOR children observed the actor performing different angles (45°, 90°, 135° and 180°) but most frequently 135° angles. Remarkably, when actively exploring the RAM, LeOS children performed mainly 45° angles (74% of their total angles), and LeOR children mainly 135° angles (26%), evidencing thus that the observational training influenced the observers to apply the main strategy they had observed. It is worth noting that the tendency to perform 45° angles is the natural explorative pattern of healthy individuals in the RAM (Mandolesi et al., 2009a). In fact, the children of all experimental groups tended to perform mainly 45° angles, although children of LeOS group performed the highest percentage of 45° angle.

On the basis of the present results it is possible to advance that behavioral units forming the strategy repertoire employed in RAM exploration can be singularly acquired through observation. The children put into action the previously observed navigational strategy significantly more frequently than the children who did not undergo any observational training (Fig. 5). In cognitive terms, this learning could be described as a priming phenomenon, which increased the activation of stored internal representations of a particular action. The primed records, now with increased salience, shaped the children’s successive exploratory behaviors. The observation of the actor’s behavior thus biased the observer’s pattern of behavior, representing a real process of observational learning.

This interpretation is in agreement with the classic theoretical framework that posits that the observational learning requires that observers understand the other’s actions in terms of the same neural code they use to produce the same motor behavior themselves (Decety & Grèzes, 1999) suggesting that the processes of learning by observation are very similar to the process of learning by doing (Petrosini, 2007).

The research on brain structures involved in observational learning advances that the mirror neuron system that is responsive to both observation and execution of action, may be differently integrated with other brain structures depending on the kind of imitative task to be performed. Namely, when observational learning is aimed at acquiring novel actions, activation of the mirror circuit may be integrated with the additional activation of the dorsolateral prefrontal cortex, an area correlated with the selection of motor acts, and with the activation of the premotor areas relevant to motor preparation (Iacoboni, 2005). In the task of the present research, the observational learning was aimed at developing efficient explorative strategies and building cognitive spatial map. Probably, besides the previously quoted cortical areas, the activation of the mirror system can be integrated with the activation of the cerebellar areas known to be implicated in procedural learning and acquisition of navigational strategies (Leggio et al., 2000; Petrosini, 2007). In this regard, it was evidenced the activation of the cerebellar areas in many forms of the “motor thought” whether or not it is accompanied by actual motor acts (Calvo-Merino, Grèzes, Glaser, Passingham, & Haggard, 2006).

We wondered whether through observation of navigational strategies, the children really built a cognitive spatial map or whether they learned to copy the observed trajectories without developing any cognitive map. The explorative behavior of the observer children was not a stereotyped copy of the behaviors previously observed: LeOS and LeOR children did not begin their exploration from the same arm explored as the first arm by the actor, they did not exhibit the same counter-clockwise or clockwise turning, and they did not exactly reproduce the sequence of entries. In short, they did not exhibit a mirror copy of the explorative behavior they had previously observed. Their performances were coherent and elaborate spatial procedures aimed at maze exploration.

Furthermore, it has to be taken into account that the children were in a different spatial position during observation and during testing, forcing them to allocentrically encode the environmental coordinates. Notably, the observation of a specific navigational strategy helped the children to build the cognitive spatial map and consequently to acquire/enrich the declarative knowledge of the environment. Because of the not yet complete functional maturation of the cerebral networks involved in spatial information processing (Overman, Pate, Moore, & Peuster, 1996; Lehnung et al., 1998; Leplow et al., 2003), the declarative spatial competence is not yet fully developed in children younger than about 7 years of age and reaches its complete development in late childhood and adolescence as the maturation of the fronto-parietal network occurs (Klingberg, 2006). Since the children to the present research were aged 5.3 years on average, the processes underlying the acquisition of cognitive spatial map were still immature. In fact, as it is typical of their age (Mandolesi et al., 2009b), the children of LeD and LeOR groups did not stop their search after collecting all rewards in a higher percentage of trials in comparison to LeOS children the majority of whom (although had the same age) stopped their exploration when they had collected the eight rewards. Interestingly, most drawings of the RAM of LeD group were characterized by an egocentric vision of the spatial context, in which the locations of the buckets were represented with respect to the particular perspective of the child. LeOR children’s drawings represented the RAM with a midway vision between the egocentric and allocentric extremes. Surprisingly, in most drawings of LeOS children the RAM was depicted with a view from above, with a clear allocentric perspective external to the child and independent of his or her position (Fig. 7). These findings indicate the capacity of the great majority of LeOS children of transforming the egocentric information acquired during exploration in allocentric representation, and they support the idea that to represent a new environment and build the cognitive spatial map a subject has to explore it appropriately (Mandolesi, Leggio, Spirito, & Petrosini, 2003).
